# Systematic review and meta-analysis of genetic association studies of pelvic organ prolapse

**DOI:** 10.1007/s00192-021-04782-2

**Published:** 2021-04-24

**Authors:** Kristina Allen-Brady, John W. F. Chua, Romana Cuffolo, Marianne Koch, Felice Sorrentino, Rufus Cartwright

**Affiliations:** 1 Department of Internal Medicine, Genetic Epidemiology, University of Utah, Salt Lake City, UT USA; 2 Zhongshan Hospital, Fudan University, Shanghai, China; 3 Department of Obstetrics & Gynaecology, John Radcliffe Hospital, Oxford University Hospitals NHS Trust, Oxford, UK; 4 Department of Obstetrics and Gynecology, Medical University of Vienna, Vienna, Austria; 5 Department of Medical and Surgical Sciences, Institute of Obstetrics and Gynecology, University of Foggia, Foggia, Italy; 6 Department of Epidemiology & Biostatistics, Imperial College London, Norfolk Place, London, UK; 7 Department of Urogynaecology, LNWH NHS Trust, London, UK

**Keywords:** Genetics, Prolapse, Meta-analysis

## Abstract

**Introduction and hypothesis:**

Family and twin studies demonstrate that pelvic organ prolapse (POP) is heritable, but the genetic etiology is poorly understood. This review aimed to identify genetic loci and specific polymorphisms associated with POP, while assessing the strength, consistency, and risk of bias among reported associations.

**Methods:**

Updating an earlier systematic review, PubMed and HuGE Navigator as well as relevant conference abstracts were searched using genetic and phenotype keywords from 2015 to 2020. Screening and data extraction were performed in duplicate. Fixed and random effects meta-analyses were conducted using co-dominant models of inheritance. We assessed credibility of pooled associations using interim Venice criteria.

**Results:**

We screened 504 new abstracts and included 46 published and 7 unpublished studies. In pooled analyses we found significant associations for four polymorphisms: rs2228480 at the *ESR1* gene (OR 0.67 95% CI 0.46–0.98, I^2^ = 0.0%, Venice rating BAB), rs12589592 at the *FBLN5* gene (OR 1.46 95% CI 1.11–1.82, I^2^ = 36.3%, Venice rating BBB), rs484389 in the *PGR* gene (OR 0.61 95% CI 0.39–0.96, I^2^ = 32.4%, Venice rating CBB), and rs1800012 at the *COL1A1* gene (OR 0.80 95% CI 0.66–0.96, I^2^ = 0.0%, Venice rating BAB). Further credible novel variants have also been recently identified in genome-wide association studies.

**Conclusion:**

The genetic contributions to POP remain poorly understood. Several biologically plausible variants have been identified, but much work is required to establish the role of these genes in the pathogenesis of POP or to establish a role for genetic testing in clinical practice.

## Introduction

The existence of inherited risk factors for pelvic floor disorders has been recognized for > 150 years [1], and multiple studies have confirmed familial aggregation of pelvic organ prolapse (POP). Three large meta-analyses demonstrated a significant impact of family history on the development of or recurrence of POP with odds ratios ranging between 1.84 to 2.64 [2–4] with an affected first-degree relative (mother or sister). Large population database studies have shown similar results. In a Swedish registry including data for 61,323 women with a history of POP surgery, the relative risk of prolapse surgery was found to be 6.58 (95% CI 6.32–6.86) for their sisters and 2.56 (2.41–2.73) for their mothers [5]. These results were further clarified in a population-based study in the USA involving 453,522 total women and 4628 women with a history of POP surgery that found that risk increased with increasing numbers of affected relatives, from RR of 2.36 (95% CI 2.15–2.58) for ≥ 1 affected first-degree relative to RR 6.26 with ≥ 3 first-degree relatives (95% CI 1.29–18.20) [6]. Having ≥ 3 affected third-degree relatives (first cousins) carried a similar risk to having one affected first-degree relative. A relevant family history is also associated with earlier onset disease [7]. Maternal inheritance of POP has been found to be a more significant contributor to the development of POP, but paternal inheritance also contributes to risk [6, 7].

Family studies, particularly those involving nuclear family members, provide limited information on heritability, as they do not control for shared exposure to environmental risk factors. Twin studies have been used to formally quantify the heritability of prolapse. In a sample of 16,886 Swedish twins aged > 50 years, heritability was estimated as 43% for prolapse surgery [8], suggesting prolapse is of similar heritability to other pelvic floor disorders including urinary incontinence.

Given the strong heritability findings, genetic studies are justified to find POP predisposition variants. Early linkage studies identified target regions that have prompted multiple follow-up candidate gene studies. The first linkage analysis investigated a single three-generation Filipino pedigree with six affected women with early-onset POP, and they identified the candidate gene *LAMC1* under their 1q31 linkage peak [9]. Two additional linkage studies involving women of European descent identified the chromosome 9q21, 10q24–26 (includes candidate gene *LOXL4*), and the 17q25 (includes candidate gene *TIMP2*) regions as showing significant evidence of linkage [10, 11]. A follow-up study involving Russian women with POP identified a significant haplotype association in the 9q21 region with results driven primarily by SNP rs12237333 [12]. These linkage analyses have been followed by multiple candidate gene studies and recently genome-wide association studies (GWAS) that are the main focus for this systematic review.

## Objective

Identification of the genetic variants underlying the heritability of POP would provide useful markers for clinical risk, prognosis, and treatment response. In addition, these insights should help explain the pathogenesis of POP, potentially offering new drug targets and preventative strategies. The aim of this systematic review was therefore to assess which polymorphisms and/or genetic loci had been tested for an association with pelvic organ prolapse in women, while assessing the strength, consistency, and potential for bias, among published associations.

## Materials and methods

### Eligibility criteria

This review updates an earlier review using the same eligibility criteria and including all prolapse studies from that work [13]. The protocol for the earlier work was prospectively registered (PROSPERO 2011:CRD42012001983), and we made no changes to the methods [14]. We pre-specified inclusion of both case-control and cross-sectional designs, with both population-based samples and other sampling methods. We included association studies testing for any genetic polymorphism at the nucleotide level, including SNPs, deletions, duplications, and copy-number variants, but excluded larger microscopic variants at the karyotype level.

There are no gold standard diagnostic methods. For pelvic organ prolapse, validated staging systems, including POP-Q, have been widely used, but again there is no universally accepted criterion for diagnosis. We therefore expected to accept diagnostic criteria for prolapse as specified within each study. In view of heterogeneity in definitions across studies, we tested for heterogeneity between studies with different criteria in different settings. We accepted definitions based on symptom questionnaires, clinical examination, or other validated assessments. We considered the population of interest as women aged ≥ 18 years.

### Search strategy

We updated the earlier systematic review, using an identical search strategy [13]. We combined searches from PubMed, HuGE Navigator, and an extensive selection of genetic, urological, and urogynaecological conference reports. In this update we searched PubMed from January 1, 2015, to November 1, 2020, using a combination of genetic and phenotype keywords and MeSH terms:

(polymorphism OR SNP OR CNV OR "copy number variation" OR mutation OR genetic OR chromosome OR VNTR OR InDel OR microsatellite) AND (prolapse OR "Pelvic Organ Prolapse"[MeSH]) NOT mitral NOT carcinoma [Title] NOT cancer [Title] NOT (animals[mh] NOT humans[mh])

In this update we searched HuGE Navigator, also from January 1, 2015, to November 1, 2020, using the phenotype indexing term *“pelvic organ prolapse.”*

In addition, we searched conference abstracts for annual meetings of the American Society of Human Genetics, American Urological Association, American Urogynecologic Society, European Association of Urology, European Society of Human Genetics, International Continence Society, International Urogynecological Association, and Society of Gynecologic Surgeons 2005–2020.

### Screening and data extraction

We developed standardized data forms for this study and conducted pilot screening and data extraction training exercises to achieve a high level of consensus between reviewers. All screening and data extraction were then performed independently and in duplicate by methodologically trained reviewers. Reviewers screened study reports by first screening titles and abstracts to select papers for full-text assessment and then screening full-text papers to confirm eligibility of the articles. Screening discrepancies were resolved by adjudication. We hand searched reference lists of all included articles, applying the same standardized screening process. When more than one report was identified for the same association in the same study population, we included the publication with the largest sample size.

We contacted study authors by email, with a reminder after 1 month, for clarifications, additional information about methodology, and additional subgroup analyses where necessary. Data extracted included information on the setting for each study, details of the sampling strategy and sampled populations (age, parity, ethnic/racial composition, and BMI), the overall sample size and proportion genotyped, the outcome assessments used and phenotypic definitions, the genotyping method employed, and the genotyping quality control applied. Where possible we extracted or requested from authors full genotype frequencies among both cases and controls.

### Statistical analysis and risk of bias assessments

For polymorphisms assessed in ≥ 2 studies for the same phenotype and evaluated with similar case definitions, we conducted fixed or random effects meta-analyses as appropriate using the Metan package (Stata 12.1). In situations where a proxy SNP had been selected for genotyping in one or more studies, in high linkage disequilibrium (defined as D′ ≥ 0.8) with another SNP of interest, these SNPs were considered as being equivalent for meta-analysis purposes; results are reported based on the original significant SNP identifier. Linkage disequilibrium was assessed between pairs of SNPs using the LDpair tool [15, 16] and an appropriate racially and ethnically matched population (e.g., Utah residents from North and West Europe [CEU] for Caucasian European populations). In all cases we worked from genotype or allele frequencies rather than using precalculated effect sizes. In the absence of a clear rationale supporting any specific model of inheritance, we used the allelic association test and co-dominant models of inheritance for all polymorphisms. We assessed the credibility of pooled associations using the interim Venice criteria [17] (see Table 1). We used the I^2^ statistic as a measure of between study heterogeneity. We recalculated the power of each study and retested for departure from Hardy-Weinberg equilibrium. We made assessments of risk of bias in phenotype definitions, genotyping, and population stratification. We used the Harbord test of funnel plot asymmetry and the significance chasing bias test [18] to investigate possible reporting biases. Throughout these assessments we used *p* < 0.05 as the criterion for significance, except in relation to GWAS, where *p* < 5 × 10^−8^ is accepted as the criterion for significance. Reporting of this review complies with recommendations of both the HuGE Handbook and the PRISMA statement.

**Table 1:** Summary of interim Venice guideline ratings of credibility of genetic associations

Criteria	Categories
Amount of evidence	**A**: Large-scale evidence (*n* > 1000 with risk allele) **B**: Moderate amount of evidence (*n* = 100–1000) **C**: Little evidence (*n* < 100)
Replication	**A**: Extensive replication including at least one well-conducted meta-analysis with little between-study inconsistency (I^2^ < 25%) **B**: Well-conducted meta-analysis with some methodological limitations or moderate between-study inconsistency (I^2^ 25%–50%) **C**: No association; no independent replication; failed replication; scattered studies; flawed meta-analysis or large inconsistency (I^2^ > 50%)
Protection from bias	**A**: Bias, if at all present, could affect the magnitude but probably not the presence of the association **B**: No obvious bias that may affect the presence of the association but there is considerable missing information on the generation of evidence **C**: Considerable potential for or demonstrable bias that can affect even the presence or absence of the association

Strong credibility for an association requires an **AAA** rating. Any **B** rating confers maximum moderate credibility, while any **C** rating confers weak credibility. Abridged from Table 4 in Ioannidis et al. [18]

### Narrative summaries

For completeness of this review, we additionally provide summaries of the four genome-wide association studies (GWAS) reported to date. Where possible, significant GWAS findings have been included in meta-analyses. However suggestive and non-significant GWAS findings are typically not reported; hence, we are unable to include most null findings from GWAS in the meta-analyses.

## Results

### Included studies

**Fig. 1:**
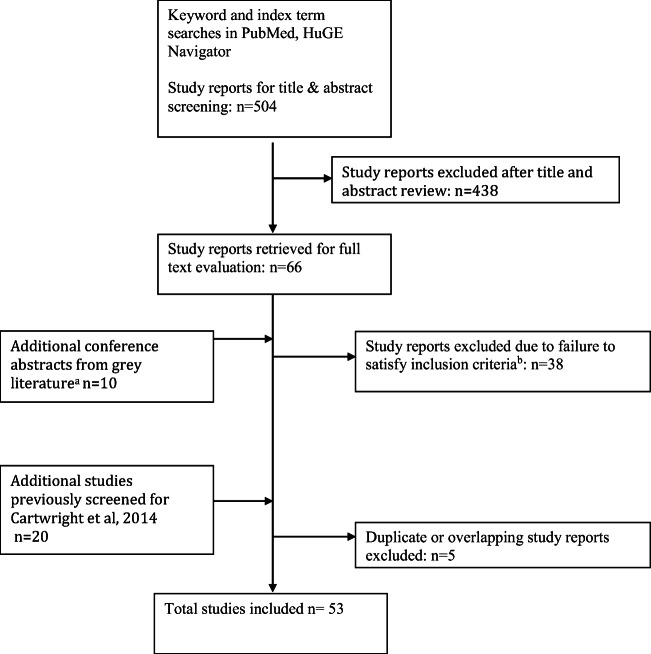
Flowchart outlining the literature search and article evaluation process. **a** ASHG, ESHG, ICS, IUGA, AUA, SGS, AUGS, and EAU abstracts 2005–2020 using search interfaces at http://www.ics.org/publications/abstracts, http://www.sciencedirect.com/science/journal/15699056, http://www.jurology.com/supplements, http://www.ashg.org/meetings/meetings_abstract_search.shtml, and/or full text search of abstract book PDFs. **b** Includes reviews (*n* = 2), inapplicable phenotypes (*n* = 3), and other study designs including pharmacogenetic studies, gene expression studies, or methylation studies (*n* = 33)

We screened 504 new abstracts for this review (Fig. 1), eventually including 46 published and 7 unpublished studies, of which 20 had been previously included in the review we updated [13]. A large majority of studies had enrolled either women of European or East Asian descent, with limited representation of other ethnicities.

### Meta-analyses

**Fig. 2:**
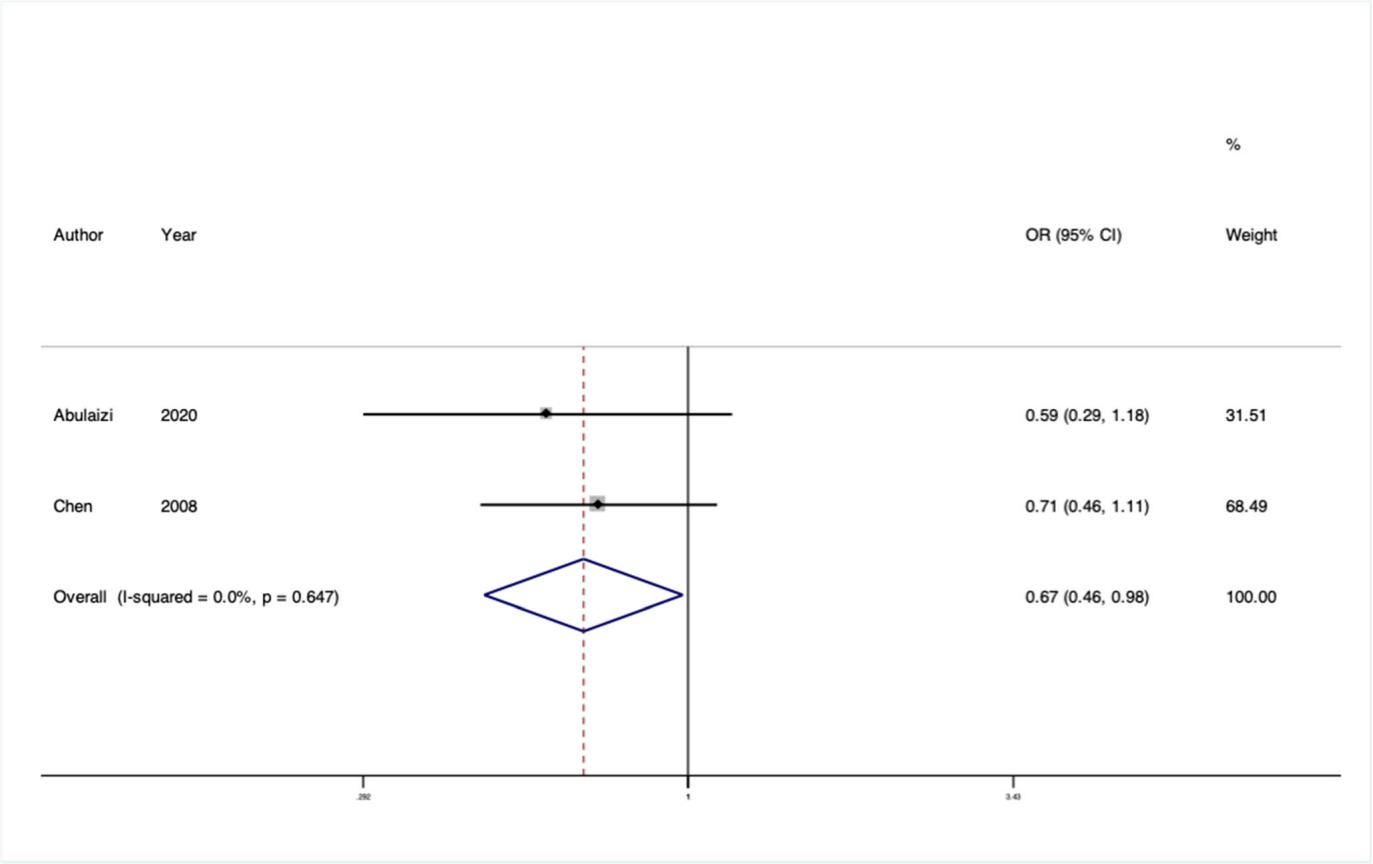
Forest plot of meta-analysis of studies of the rs2228480 SNP in the gene *ESR1*

**Fig. 3:**
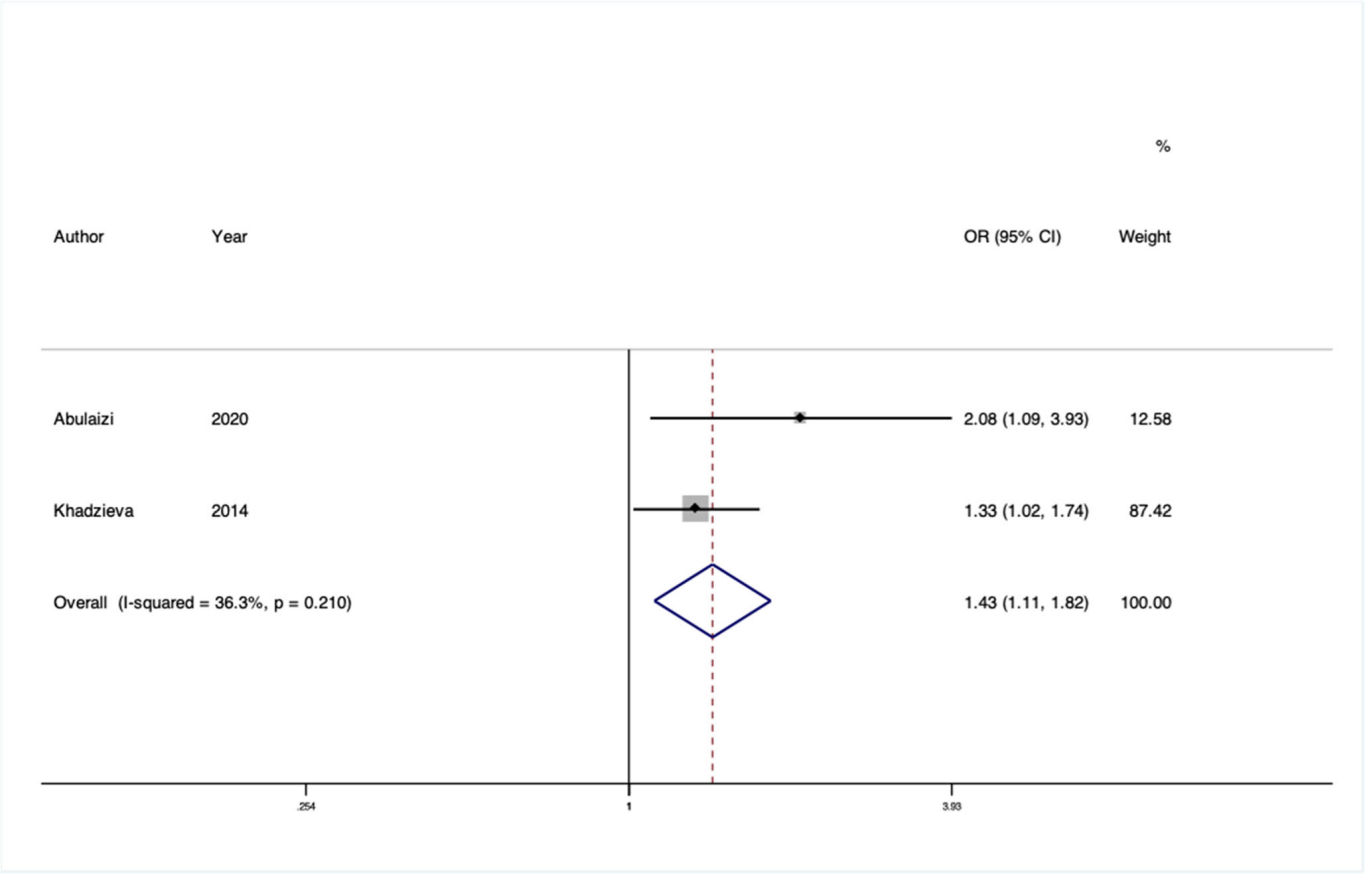
Forest plot of meta-analysis of studies of the rs12589592 SNP in the gene *FBLN5*

**Fig. 4:**
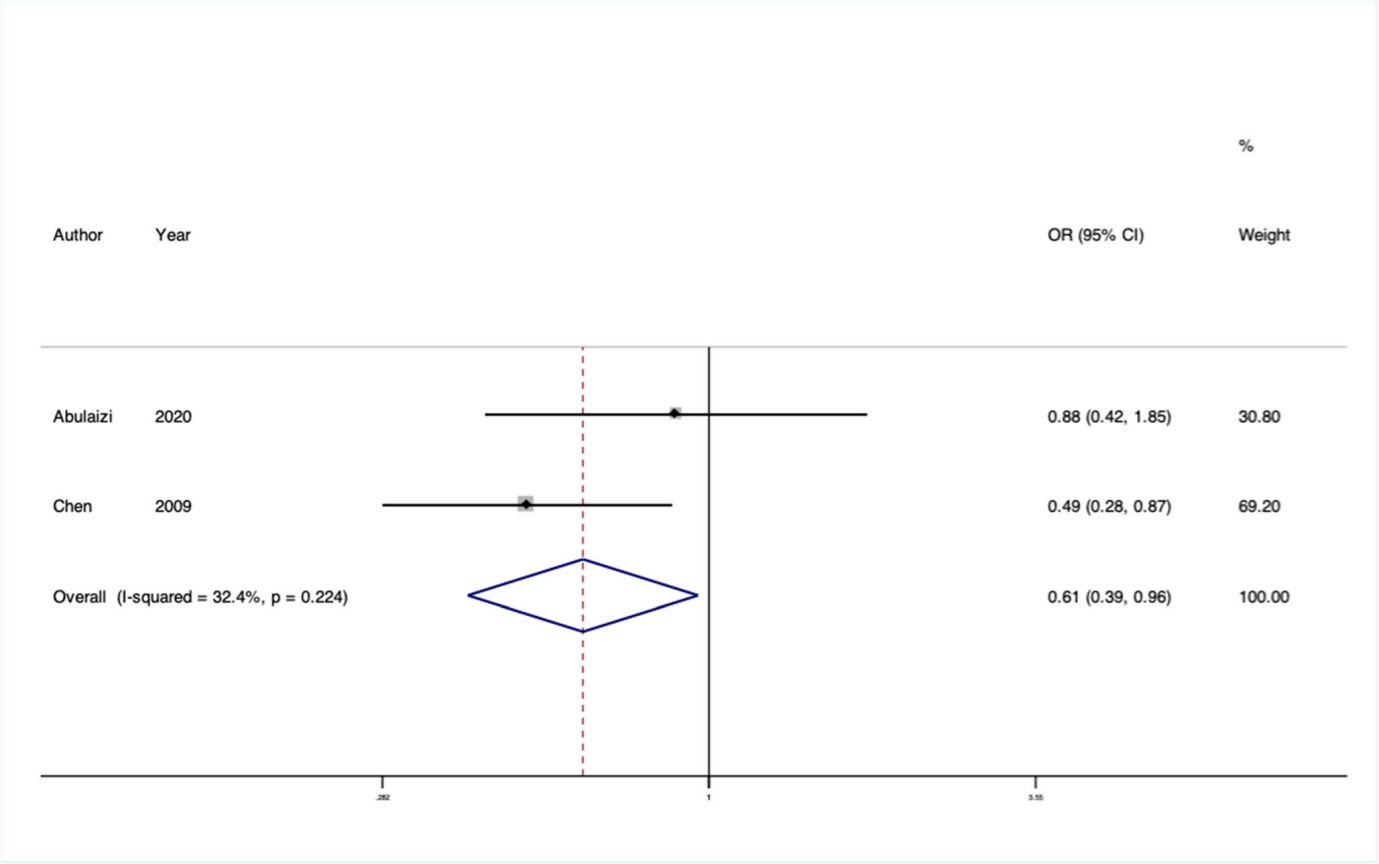
Forest plot of meta-analysis of studies of the rs484389 SNP in the gene *PGR*

**Fig. 5:**
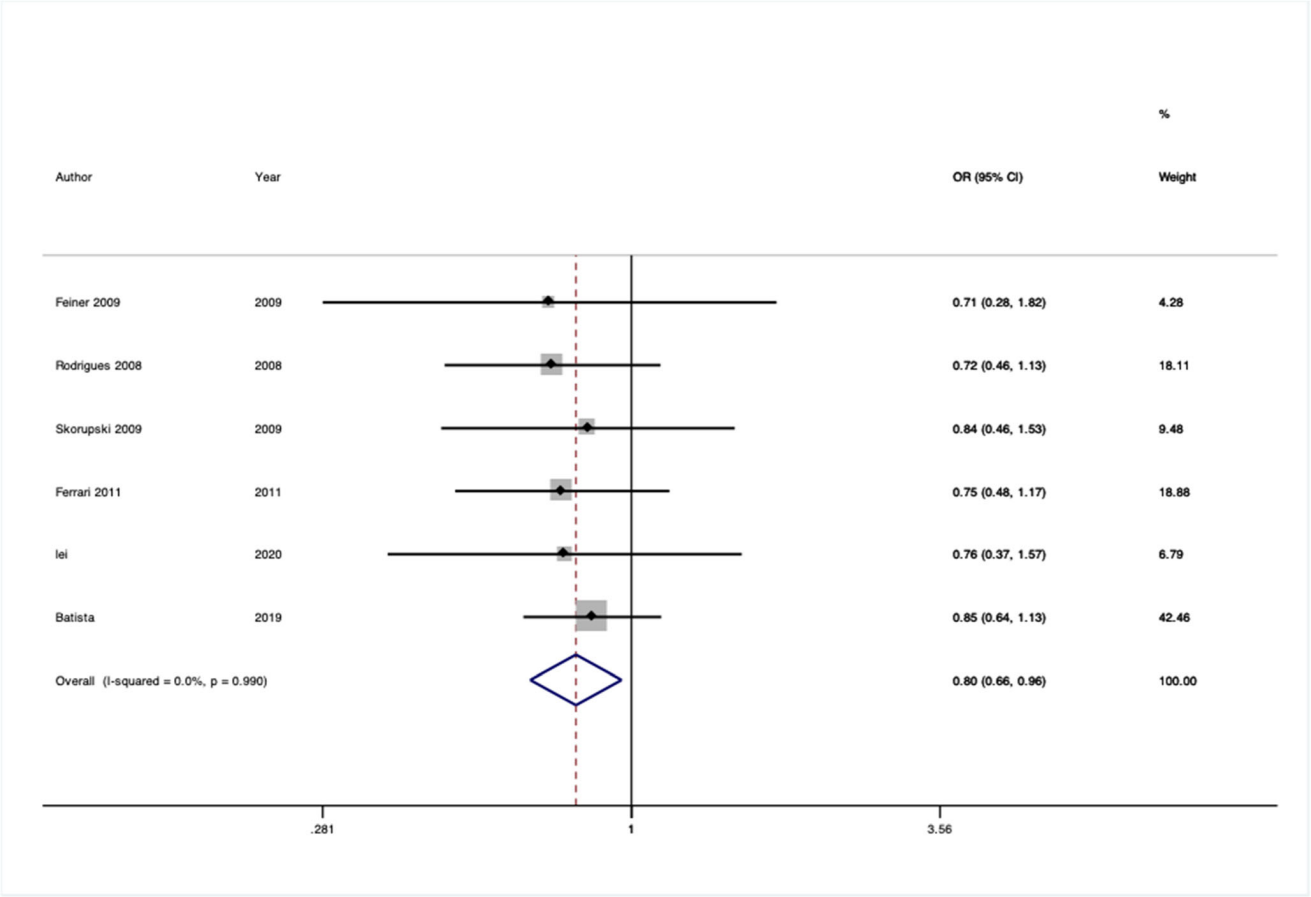
Forest plot of meta-analysis of studies of the rs1800012 SNP in the gene *COL1A1*

We conducted 24 separate meta-analyses for variants in or near 16 different genes or genetic loci. Four of these 12 genes had significant findings in pooled analyses: rs2228480 in the *ESR1* gene, rs12589592 in the *FBLN5* gene, rs484389 in the *PGR* gene, and rs1800012 in the *COL1A1* gene (Figs. 2, 3, 4, and 5).

#### ESR1 gene

*ESR1* is an estrogen receptor gene, which was identified as relevant in candidate gene studies because of the epidemiological association between estrogen status and prolapse. Two studies from Taiwan and China assessed the same three variants (rs17847075, rs2228480, and rs2234693) and could be included in meta-analyses [19, 20]. In pooled analyses, rs2228480 showed a large protective effect with low heterogeneity (OR = 0.67, 95% CI: 0.46–0.98, I^2^ = 0.0%, Venice rating BAB). The risk variant is common in the populations assessed, and so despite the low total sample size (*n* = 339), this confers moderate epidemiological credibility.

#### FBLN5 gene

*FBLN5* has been investigated as a candidate gene for prolapse as fibulins play a critical role in the assembly of elastic fibers, believed to provide strength and flexibility in the pelvic floor. Three studies from Brazil, Russia, and China assessed the same two variants (rs2018736 and rs12589592) of which two studies could be included in meta-analyses [19, 21, 22]. No significant pooled effect was observed for rs2018736, but a large effect was seen at rs12589592 with moderate heterogeneity (OR 1.43 95% CI 1.11–1.82, I^2^ = 36.3%, Venice rating BBB). The risk variant is common in the populations assessed, and so despite the low total sample size (*n* = 568), this confers moderate epidemiological credibility.

#### PGR gene

*PGR* has been investigated as a candidate gene for prolapse, as it codes for the progesterone receptor, and changes in serum progesterone cyclically, during pregnancy, and after menopause are all observed to have an influence on prolapse. Two studies from China each assessed the same two common polymorphisms and could be included in meta-analyses [19, 23]. No significant pooled effect was observed for rs500760, but a large effect was seen at rs484389 with moderate heterogeneity (OR = 0.61, 95% CI: 0.39–0.96, I^2^ = 32.4%, Venice rating CBB). The risk variant is common in the populations assessed, but the low total sample size (*n* = 336) confers weak epidemiological credibility.

#### COL1A1 gene

*COL1A1* has been investigated as a candidate gene for prolapse as it forms type 1 collagen, the most abundant human collagen. The rs1800012 was identified as a replicated locus in our earlier review, but we could now include six studies with a moderate protective effect with no heterogeneity (OR = 0.80, 95% CI: 0.66–0.96, I^2^ = 0.0%, Venice rating BAB) [24–28]. The risk variant is common in the populations assessed, and with a moderate sample size (*n* = 1264), this confers moderate epidemiological credibility.

#### Other genes

We conducted further meta-analyses for variants in *COL3A1* type 3 collagen (8 studies), *COL18A1* collagen type 18 (3 studies), *LAMC1* Laminin, gamma 1 (6 studies), *ZFAT* (3 studies), *MMP1* matrix metalloproteinase 1 (3 studies), *MMP3* matrix metalloproteinase 3 (4 studies), *MMP9* matrix metalloproteinase 9 (4 studies), *MMP10* matrix metalloproteinase 10 (2 studies), and four other variants identified from GWAS (rs1455311, rs430794, rs8027714, and rs1810636). None of these meta-analyses showed significant pooled effects. Results are summarized in Table 3. Many genes had been assessed in a single study only and as such require replication for credibility (Table 2).

**Table 2:** Included studies

First author	Journal & year	Country	Descent/ethnicity/race^a^	Gene symbols(s)	Polymorphism(s) dbSNP ID	Case definition	Control definition	n Cases genotyped	n Controls genotyped
Abulaizi [19]	Int Urogynecol J 2020	China	Mixed Chinese	*ESR1* *ESRB* *ZFAT* *FBLN5* *PGR* *COL3A1* *MMP9* *LAMC1*	rs17847075 rs2234693 rs2228480 rs1271572 rs2987983 rs1256049 rs484389 rs500760 rs1800255 rs391253 rs17576 rs1036819 rs10911193 rs20563 rs2018736 rs12589592	POP ≥ stage 3	POP stage 0 or 1	88	108
Allen-Brady [29]	Obstet Gynecol 2011	USA Netherlands	White and Northern European descent	*LINC0108*^*b*^*8* *ZFAT* Intergenic Intergenic Intergenic *COL18A1*	rs1455311 rs1036819 rs430794 rs8027714 rs1810636 rs2236479	Surgically treated/recurrent POP with family history	Illumina iControlDB and HapMap Utah population controls	191	3036
Ashikari [37]	Neurourol Urodyn 2019 (ICS abstract)	Japan	Japanese	*COL3A1*	rs1800255	POP ≥ stage 3	POP stage 0 or 1	40	17
Campeau [38]	Neurourol Urodyn 2011 (ICS Abstract)	USA	Not stated	*MMP1*	rs1144393 rs498186 rs473509	Surgically treated POP	Hospital controls “without POP”	63	93
Batista [39]	Neurourol Urodyn	Brazil	Brazilian	*COL1A1* *COL3A1*	rs1800012 rs1800255	POP ≥ stage 3	POP stage 0 or 1	348	286
Bizjak [31]	Eur J Obs Gyn 2020	Slovenia	White	*LINC0108*^*b*^*8* *ZFAT* Intergenic Intergenic Intergenic *COL18A1*	*rs6852257* *rs1036819* *rs4436246* *rs77662161* *rs6051098* *rs72794445*	Surgical repair stage III-IV uterine POP; age 30–55 years	Consecutive volunteers with intact pelvic support	118	114
Chen [40]	Am J Obstet Gynecol 2010	USA	African American and Caucasian	*LAMC1*	rs10911193 rs20563 rs20558	POP > stage 2	POP < stage 2	165	246
Chen [20]	Int Urogynecol J 2008	Taiwan	Taiwanese	*ESR1*	rs17847075 rs2207647 rs2234693 rs3798577 rs2228480	POPQ≥2	POPQ<2	88	153
Chen [41]	Int J Clin Exp Pathol 2015	China	Han Chinese	*RAGE*	rs184003 rs55640627	POP ≥ stage 3	POP stage 0 or 1	24	25
Chen [23]	Acta Obs Gyn 2009	Taiwan	Taiwanese	PGR	rs500760 rs484389	POPQ≥2	POPQ<2	87	150
Chen [42]	Int Urogynecol J 2008	Taiwan	Taiwanese	*COL3a1*	rs1800255 rs1801184	POPQ≥2	POPQ<2	84	147
Chen [43]	Eur J Obs Gyn 2010	Taiwan	Taiwanese	*MMP9*	rs3918242 rs17576 rs2250889	POPQ≥2	POPQ<2	92	152
Chen [44]	Eur J Obs Gyn 2008	Taiwan	Taiwanese	*ESR2*	rs2987983 rs1271572 rs944459 rs1256049 rs1255998	POPQ≥2	POPQ<2	69	141
Chen [45]	Hereditas 2020	China	Chinese	*LAMC1*	rs20558 rs20563 rs10911193 rs6424889 rs10911241 rs3768617 rs12073936 rs729819 rs10911214 rs869133	POP stage III or IV	POP stage 0 or I	161	235
Cho [24]	Yonsei Med J 2009	Korea	Korean	*COL1A1*	rs1800012	Surgically treated POPQ≥3	POPQ = 0	15	15
Choy [46]	Neurourol Urodyn 2007 (ICS Abstract)	Hong Kong	Chinese	*EDN1*	rs5370 rs10478694	POPQ≥2	Hospital “normal” controls and HapMap Han Chinese controls	60 (rs5370) and 67 (rs10478694)	210
de Paula [22]	Rev Assoc Med Bras 2020	Brazil	Brazilian	*FBLN5*	rs12586948	POP stage III or IV	POP stage 0 or 1	112	180
dos Santos [32]	Int Urogynecol J 2018	Brazil	Brazilian	*COL18A1* *LOXL4*	rs2236479 rs2862296	POP ≥ stage 3	POP stage 0 or 1	285	247
Feiner [25]	Int Urogynecol J 2009	Israel	Caucasian or Ashkenazi-Jewish	*COL1a1*	rs1800012	POPQ≥3	POPQ<2	36	36
Ferrari [27]	Arch Gynecol Obstet 2012	Italy	Italian	*COL1a1* *MMP9* *MMP1* *MMP3*	rs1800012 rs3918242 rs1799750 rs3025058	POPQ≥2	POPQ<2	137	96
Ferrell [47]	Reprod Sci 2009	USA	African American or Caucasian	*LOXL1*	rs16958477	POP ≥ stage II	POP < stage II	137	130
Fu [48]	J Urol 2009 (AUA Abstract)	USA	Not stated	*LAMC1* *LOXL1*	rs10911193	POP ≥ stage III	No POP or UI	61	33
Giri [36]	PLOS ONE 2015	USA	African American and Hispanic	*ABCA1* *FHAD1* *ANKS4B* *MAML2*	rs7035589 rs139563135 rs144039930 rs10160713	POP ≥ stage I	POP stage 0	1399	1253
Ghersel [49]	Rev Bras Ginecol Obstet 2019	Brazil	Brazilian	*MMP9*	rs3918242	POP ≥ stage 3	POP stage 0 or 1	86	158
Jeon [50]	J Urol 2009	Korea	Korean	*COL3a1*	rs111929073	POPQ≥2	POPQ<2 and no USI	36	36
Karachalios [51]	Biomed Rep 2016	Greece	White	*MMP3*	rs3025058	POPQ≥2	POPQ<2	80	80
Kasyan [52]	Urologiia 2017	Russia	White	*COL3A1*	rs1800255	POP and UI	No PFD	52	21
Khadzhieva [21]	Maturitas 2014	Russia	White	*FBLN5*	rs2430339 rs929608 rs12586948 rs2284337 rs2430347 rs2498841 rs2018736 rs12589592 rs2430369 rs2245701 rs2474028	POP ≥ stage III	POP stage 0	210	292
Khadzhieva [53]	Genetika 2015	Russia	White	*FBLN3* *LOXL1*	rs2165241 rs2304719 rs893821 rs3791679 rs1367228 rs3791660 rs2033316	POP ≥ stage III	POP stage 0	210	292
Khadzhieva [12]	Biomed Res Int 2015	Russia	White	*LINC01088* *ZFAT* *COL18A1* *TLE4* *TLE1* *LOC102723989* *FRMD3* *COL18A1*	rs1455311 rs1036819 rs4077632 rs2807303 rs2777781 rs11139451 rs12237222 rs12551710 rs430794 rs8027714 rs1810636 rs2236479	POP ≥ stage III	POP stage 0	210	292
Kieserman-Shmokler [35]	Int Urogynae J 2019	USA	European	*NPAP1* *GDF7* *SALL1*	rs8027714 rs12325192 rs9306894				
Kim [54]	Euro J Obstet Gynecol Repro Biol 2014	Korea	Korean	*GSTM1* *GSTT1* *GSTP1*	Null Null rs1695	POPQ≥3	POPQ<2	189	156
Kim [55]	Menopause 2014	Korea	Korean	*PARP1*	rs1136410	POPQ≥3	POPQ<2	185	155
Li [33]	Menopause 2020	China	Chinese	*COL14A1* *COL5A1* *COL4A2* *COL3A1* *COL1A1* *COL18A1*	rs4870723 rs2305600 rs2305598 rs2305603 rs3827852 rs445348 rs76425569 rs388222 rs2281968 rs74941798 rs2586488	POP ≥ stage 3	POP stage 0	48	48
Lince [56]	Int Urogynecol J 2014	The Netherlands	≈ 99% Dutch	*COL3a1*	rs1800255	POPQ≥2	POPQ<2	272	82
Maeda [57]	Euro J Obstet Gynecol Repro Biol 2019	Brazil	White or non- white	*MMP3*	rs3025058	POP ≥ stage 3	POP stage 0 or 1	112	180
Martins [58]	Neurourol Urodyn 2011	Brazil	White or non- white	*COL3a1*	rs111929073	POP ≥ stage III	POP < stage II	107	209
Nakad [59]	Taiwan J Obstet Gynecol 2017	Taiwan	Taiwanese	*ESRA* *LAMC1*	rs10911193 rs2228480	POP ≥ stage 3	POP stage 0 or 1	33	33
Neupane [60]	Female Pelvic Med Reconstr Surg 2014	USA		*LOXL1*	rs1048661 rs3825942 rs78803776 rs41429348 rs41435250 rs369758147	POP ≥ stage 3	POP stage 0 or 1	48	18
Olafsdottir [34]	Commun Biol 2020	Iceland/UK	White	*WNT4* *GDF7* *EFEMP1* *FAT4* *IMPDH1* *TBX5* *SALL1*	rs3820282 rs9306894 rs3791675 rs7682992 rs1247943 rs12325192 rs72624976 rs1430191	ICD 9/10 codes indicating POP	Unselected female population controls	15,010	340,734
Palos [61]	Int Urogynecol J 2020	Brazil	White or non- white	*COL1a1*	rs1107946	POP ≥ stage 3	POP stage 0 or 1	112	180
Rao [62]	PLOS ONE 2015	China	Han Chinese	*WNK1*	Novel variants	POP ≥ stage III	Healthy post-menopausal	161	231
Rodrigues [26]	Int Urogynecol J 2008	Brazil	White or non- white	*COL1a1*	rs1800012	POP ≥ stage III	POP < stage II and no SUI	107	209
Romero [63]	J Pelv Med Surg 2008	USA	White	*MMP1* *MMP2* *MMP3* *MMP8* *MMP9* *MMP10* *MMP11* *TIMP1* *TIMP3*	rs2071230 rs7201 rs679620 rs35866072 rs17576 rs17435959 rs738789 rs4898 rs2016293	POPQ ≥ 3	POPQ<2 and no UI	45	38
Rosa [64]	Rev Bras Ginecol Obstet 2019	Brazil	White or non- white	*COL1A2*	rs42524	POP ≥ stage 3	POP stage 0 or 1	112	180
Rusina [65]	Neurourol Urodyn 2014 (ICS Abstract)	Russia	White	*NAT2* *GSTT1* *GSTM1*	rs1799929 rs1799931 Null Null	POP ≥ stage I	POP stage 0 and no UI	63	89
Skorupski [28]	Int Urogynecol J 2009 (IUGA abstract)	Poland	Polish	*COL1a1*	rs1800012	POPQ ≥ 2	POPQ<2 and no UI	120	97
Skorupski [66, 67]	Ginekol Polska 2010/Int Urogynecol J 2013	Poland	Polish	*MMP1* *MMP3*	rs1799750 rs3025058	POPQ ≥ 2	POPQ<2	132	133
Teixeira [68]	Int Urogynecol J 2020	Brazil	White or non- white	*COL3A1*	rs1800255	POP ≥ stage 3	POP stage 0 or 1	112	180
Vishawajit [69]	Neurourol Urodyn	USA	Not stated	*MMP1*	rs1799750	Unclear	Unclear	40	15
Wang [70]	J Obstet Gynaecol Res. 2015	China	Chinese	*MMP10*	rs17435959 rs17293607	Unclear	Unclear	91	172
Wu [71]	Am J Obstet Gynecol 2012	USA	Non-Hispanic white	*LAMC1*	rs10911193 rs1413390 rs20558 rs20563 rs10911206 rs2296291 rs12041030 rs12739316 rs3768617 rs2483675 rs10911211 rs41475048 rs1058177 rs12073936	POPQ ≥ 3	POPQ<2	239	197
Wu	Obstet Gynecol 2012	USA	Non-Hispanic white	*MMP9*	rs3918253 rs3918256 rs3918278 rs17576 rs2274755 rs17577 rs2236416 rs3787268	POPQ ≥ 3	POPQ<2	239	197

^a^Assessments of descent/ethnicity/race as specified in primary publications, or from additional data from authors, or assumed for countries with low ethnic heterogeneity including Taiwan, Korea, and Japan

### Narrative summary of GWASes

The first GWAS for POP involved 115 surgically treated, related POP cases who were part of high-risk POP pedigrees and 2976 population-based controls [29]. They identified six variants at chromosomal regions 4q21 (rs1455311), 8q24 (rs1036819), 9q22 (rs430794), 15q11 (rs8027714), 20p13 (rs1810636), and 21q22 (rs2236479). Five of these six SNPs have subsequently been identified as at risk of genotyping error on one or more Illumina arrays, which may have led to spurious association signals [30]. The original study observed nominally or trending towards significance for some variants in a Dutch validation cohort of 76 POP cases. Subsequent independent replication studies [31–33, 12, 34, 19, 35] have tested for association at some or all of these six SNPs, with rs1036819 close to *ZFAT* replicating in one study [19], rs8027714 on chromosome 15q11 replicating in another study [35], and rs1810636 on chromosome 20p13, demonstrating replication in another study [31], but with no overall significant replication for any SNP observed in our meta-analyses (see Table 3).

**Table 3:** Summary of meta-analyses

Gene symbols(s)	Polymorphism dbSNP ID	n studies	n participants	Pooled OR	95% CI	p	I^2^
*ESR1*	rs17847075	2	340	0.90	0.55–1.47	0.68	51.6%
	rs2228480	2	339	0.67	0.46–0.98	**0.04**	0.0%
	rs2234693	2	339	0.93	0.67–1.27	0.63	0.0%
*ZFAT*	rs1036819	3	804	0.78	0.42–1.12	0.15	45.7%
*FBLN5*	rs2018736	2	543	0.97	0.46–2.06	0.94	82.4%
	rs12589592	2	568	1.46	1.11–1.82	**0.005**	36.3%
*LINC01088*	rs1455311	2	699	1.01	0.77–1.34	0.93	75.2%
*LOC100507103*	rs430794	2	704	1.21	0.95–1.545	0.12	0.0%
*NPAP1*	rs8027714	2	705	0.93	0.50–1.73	0.82	44.8%
*LOC105372507*	rs1810636	2	698	1.03	0.82–1.29	0.82	75.8%
*PGR*	rs484389	2	336	0.61	0.39–0.96	**0.03**	32.4%
	rs500760	2	337	1.04	0.70–1.53	0.86	0.0%
*COL3A1*	rs1800255	7	1795	1.01	0.87–1.18	0.86	0.0%
	rs111929073	2	385	0.99	0.81–1.21	0.93	0.0%
*MMP9*	rs3918278	4	1159	1.24	0.70–219	0.46	65.4%
	rs17576	4	809	0.98	0.67–1.41	0.89	58.2%
*LAMC1*	rs10911193	6	1830	1.08	0.89–1.33	0.43	0.0%
	rs20563	4	1272	1.08	0.92–1.27	0.69	0.0%
	rs20558	4	1179	1.15	0.97–1.35	0.11	0.0%
*COL18A1*	rs2236479	4	1112	1.01	0.81–1.90	0.93	32.2%
*MMP1*	rs1799750	3	601	0.82	0.64–1.04	0.10	25.1%
*COL1A1*	rs1800012	6	1264	0.80	0.66–0.96	**0.02**	0.0%
*MMP3*	rs3025058	4	925	0.96	0.79–1.15	0.67	0.0%
*MMP10*	rs17435959	2	305	2.42	0.55–10.8	0.25	37.1%

A further GWAS using African American and Hispanic women from the Women’s Health Initiative Hormone Therapy study [36] included 1427 cases with any diagnosis of POP (grades 1–3) and 317 cases diagnosed with moderate/severe POP (grades 2–3) and 1274 controls without POP (grade 0). Although they did not identify any variants meeting genome-wide significance, they did identify a number of variants that met *p* < 10^−6^.

The largest POP meta-analysis of two GWA studies involved 3409 cases from Iceland and 131,444 controls and 11,601 cases and 209,288 controls from UK Biobank, all of which were of European ancestry [34]. POP cases were identified based on ICD 9/10 coding therefore representing women who had presented for care. They identified eight variants at seven loci meeting the genome-wide significance criterion in the meta-analysis with results driven mainly by UK Biobank data. The significant SNPs include rs3820282, rs9306894, rs3791675, rs7682992, rs1247943, rs12325192, rs72624976, and rs1430191. None of the lead POP variants were coding or in high linkage disequilibrium (LD) with coding variants. We can consider them each as having moderate credibility (Venice rating ABB). This study did not replicate any variants identified by earlier GWASes [29, 36] Table 4.

**Table 4:** Interim Venice ratings of the credibility of replicated associations

Gene symbols(s)	Polymorphism dbSNP ID	Pooled OR	95% CI	I^2^	Venice rating	Overall credibility
*ESR1*	rs2228480	0.67	0.46–0.98	0.0%	BAB	Moderate
*FBLN5*	rs12589592	1.46	1.11–1.82	36.3%	BBB	Moderate
*PGR*	rs484389	0.61	0.39–0.96	32.4%	CBB	Weak
*COL1A1*	rs1800012	0.80	0.66–0.96	0.0%	BAB	Moderate

Finally, a recently reported GWAS utilizing 1329 women with diagnosed and/or surgically treated prolapse and 16,383 hospital controls did not identify any variants meeting genome-wide significance [35]. However, testing associations from previous GWASes showed nominal replication for rs8027714 [29] and for rs12325192, and rs9306894 [34].

## Conclusions

Given current evidence supporting a genetic predisposition for pelvic organ prolapse, we have identified four variants through meta-analysis of candidate gene studies significantly associated with POP (rs2228480 in the *ESR1* gene, rs12589592 in the *FBLN5* gene, rs484389 in the *PGR* gene, and rs1800012 in the *COL1A1* gene). In each meta-analysis we have at most moderate evidence in support of an association with POP. A much larger, recent prospective meta-analysis of two genome-wide association studies has identified eight variants significantly associated with POP [34], with recent evidence of replication for two of these variants in an independent population [35]. As the sizes of GWAS meta-analyses grow, further novel variants are likely to be identified providing novel insights into pathogenesis. Given the impact of pelvic floor disorders on women’s health, additional work needs to be done to provide further validation of POP predisposition variants in a variety of different populations to establish the role of these genes in the pathogenesis of prolapse and to establish a possible role for genetic testing in clinical practice that could improve patients’ outcomes and address the best treatment options.

## References

[1] Thompson H (1854). On irritability of the bladder. Lancet.

[2] Friedman T, Eslick GD, Dietz HP (2018). Risk factors for prolapse recurrence: systematic review and meta-analysis. Int Urogynecol J.

[3] Lince SL, van Kempen LC, Vierhout ME, Kluivers KB (2012). A systematic review of clinical studies on hereditary factors in pelvic organ prolapse. Int Urogynecol J.

[4] Samimi P, Jones SH, Giri A. Family history and pelvic organ prolapse: a systematic review and meta-analysis. Int Urogynecol J. ePublication ahead of print. 2020. 10.1007/s00192-020-04559-z. 10.1007/s00192-020-04559-z PMC8086380 33084962

[5] Hamer MA, Persson J (2013). Familial predisposition to pelvic floor dysfunction: prolapse and incontinence surgery among family members and its relationship with age or parity in a Swedish population. Eur J Obstet Gynecol Reprod Biol.

[6] Allen-Brady K, Norton PA, Hill AJ, Rowe K, Cannon-Albright LA (2020). Risk of pelvic organ prolapse treatment based on extended family history. Am J Obstet Gynecol.

[7] Jack GS, Nikolova G, Vilain E, Raz S, Rodriguez LV (2006). Familial transmission of genitovaginal prolapse. Int Urogynecol J Pelvic Floor Dysfunct.

[8] Altman D, Forsman M, Falconer C, Lichtenstein P (2008). Genetic influence on stress urinary incontinence and pelvic organ prolapse. Eur Urol.

[9] Nikolova G, Lee H, Berkovitz S, Nelson S, Sinsheimer J, Vilain E, Rodriguez LV (2007). Sequence variant in the laminin gamma1 (LAMC1) gene associated with familial pelvic organ prolapse. Hum Genet.

[10] Allen-Brady K, Cannon-Albright LA, Farnham JM, Norton PA (2015). Evidence for pelvic organ prolapse predisposition genes on chromosomes 10 and 17. Am J Obstet Gynecol.

[11] Allen-Brady K, Norton PA, Farnham JM, Teerlink C, Cannon-Albright LA (2009). Significant linkage evidence for a predisposition gene for pelvic floor disorders on chromosome 9q21. Am J Hum Genet.

[12] Khadzhieva MB, Kolobkov DS, Kamoeva SV, Ivanova AV, Abilev SK, Salnikova LE (2015). Verification of the chromosome region 9q21 association with pelvic organ prolapse using RegulomeDB annotations. Biomed Res Int.

[13] Cartwright R, Kirby AC, Tikkinen KA, Mangera A, Thiagamoorthy G, Rajan P, Pesonen J, Ambrose C, Gonzalez-Maffe J, Bennett P, Palmer T, Walley A, Jarvelin MR, Chapple C, Khullar V (2015). Systematic review and metaanalysis of genetic association studies of urinary symptoms and prolapse in women. Am J Obstet Gynecol.

[14] PROSPERO: A systematic review of candidate gene association studies of lower urinary tract symptoms and pelvic organ prolapse in women. http://www.crd.york.ac.uk/prospero/display_record.asp?ID=CRD42012001983#.Uk701BY4Qts. Accessed 4 October 2013.

[15] Genetics NCIDoCE LDpair Tool. https://ldlink.nci.nih.gov/?tab=home. Accessed 1 September 2020.

[16] Machiela MJ, Chanock SJ (2015). LDlink: a web-based application for exploring population-specific haplotype structure and linking correlated alleles of possible functional variants. Bioinformatics.

[17] Ioannidis JP, Boffetta P, Little J, O'Brien TR, Uitterlinden AG, Vineis P, Balding DJ, Chokkalingam A, Dolan SM, Flanders WD, Higgins JP, McCarthy MI, McDermott DH, Page GP, Rebbeck TR, Seminara D, Khoury MJ (2008). Assessment of cumulative evidence on genetic associations: interim guidelines. Int J Epidemiol.

[18] Ioannidis JP, Trikalinos TA (2007). An exploratory test for an excess of significant findings. Clin Trials.

[19] Abulaizi A, Abula A, Ababaikeli G (2020). Identification of pelvic organ prolapse risk susceptibility gene SNP locus in Xinjiang women. Int Urogynecol J.

[20] Chen HY, Chung YW, Lin WY, Chen WC, Tsai FJ, Tsai CH (2008). Estrogen receptor alpha polymorphism is associated with pelvic organ prolapse risk. Int Urogynecol J Pelvic Floor Dysfunct.

[21] Khadzhieva MB, Kamoeva S, Chumachenko AG, Ivanova AV, Volodin IV, Vladimirov IS, Abilev SK, Salnikova LE (2014). Fibulin-5 (FBLN5) gene polymorphism is associated with pelvic organ prolapse. Maturitas.

[22] Paula MVB, Lira Junior MAF, Monteiro V, Souto RP, Fernandes CE (1992). Oliveira E (2020) evaluation of the fibulin 5 gene polymorphism as a factor related to the occurrence of pelvic organ prolapse. Rev Assoc Med Bras.

[23] Chen HY, Chung YW, Lin WY, Chen WC, Tsai FJ, Tsai CH (2009). Progesterone receptor polymorphism is associated with pelvic organ prolapse risk. Acta Obstet Gynecol Scand.

[24] Cho HJ, Jung HJ, Kim SK, Choi JR, Cho NH, Bai SW (2009). Polymorphism of a COLIA1 gene Sp1 binding site in Korean women with pelvic organ prolapse. Yonsei Med J.

[25] Feiner B, Fares F, Azam N, Auslender R, David M, Abramov Y (2009). Does COLIA1 SP1-binding site polymorphism predispose women to pelvic organ prolapse?. Int Urogynecol J Pelvic Floor Dysfunct.

[26] Rodrigues AM, Girao MJ, da Silva ID, Sartori MG, Martins Kde F, Castro Rde A (2008). COL1A1 Sp1-binding site polymorphism as a risk factor for genital prolapse. Int Urogynecol J Pelvic Floor Dysfunct.

[27] Ferrari MM, Rossi G, Biondi ML, Vigano P, Dell'utri C, Meschia M (2012). Type I collagen and matrix metalloproteinase 1, 3 and 9 gene polymorphisms in the predisposition to pelvic organ prolapse. Arch Gynecol Obstet.

[28] Skorupski P (2009). Does polymorphism of the gene encoding alpha-1 chain of collagen type 1 influence the risk of pelvic organ prolapse?. Int Urogyncol J.

[29] Allen-Brady K, Cannon-Albright L, Farnham JM, Teerlink C, Vierhout ME, van Kempen LC, Kluivers KB, Norton PA (2011). Identification of six loci associated with pelvic organ prolapse using genome-wide association analysis. Obstet Gynecol.

[30] Mitry D, Campbell H, Charteris DG, Fleck BW, Tenesa A, Dunlop MG, Hayward C, Wright AF, Vitart V (2011). SNP mistyping in genotyping arrays--an important cause of spurious association in case-control studies. Genet Epidemiol.

[31] Bizjak T, Gorenjak M, Potocnik U, But I (2020). Polymorphism on chromosome 20p13 near the IDH3B gene is associated with uterine prolapse. Eur J Obstet Gynecol Reprod Biol.

[32] Dos Santos RGM, Pepicelli FCA, Batista NC, de Carvalho CV, Bortolini MAT, Castro RA (2018). Collagen XVIII and LOXL-4 polymorphisms in women with and without advanced pelvic organ prolapse. Int Urogynecol J.

[33] Li L, Sun Z, Chen J, Zhang Y, Shi H, Zhu L (2020). Genetic polymorphisms in collagen-related genes are associated with pelvic organ prolapse. Menopause.

[34] Olafsdottir T, Thorleifsson G, Sulem P, Stefansson OA, Medek H, Olafsson K, Ingthorsson O, Gudmundsson V, Jonsdottir I, Halldorsson GH, Kristjansson RP, Frigge ML, Stefansdottir L, Sigurdsson JK, Oddsson A, Sigurdsson A, Eggertsson HP, Melsted P, Halldorsson BV, Lund SH, Styrkarsdottir U, Steinthorsdottir V, Gudmundsson J, Holm H, Tragante V, Asselbergs FW, Thorsteinsdottir U, Gudbjartsson DF, Jonsdottir K, Rafnar T, Stefansson K (2020). Genome-wide association identifies seven loci for pelvic organ prolapse in Iceland and the UK biobank. Commun Biol.

[35] Kieserman-Shmokler C, Pandit A, Zawistowski M, Swenson CW (2019). Genome wide assocation study (GWAS) of pelvic organ prolapse using an institutional genomics initiative. Female Pelvic Med Reconstr Surg.

[36] Giri A, Wu JM, Ward RM, Hartmann KE, Park AJ, North KE, Graff M, Wallace RB, Bareh G, Qi L, O'Sullivan MJ, Reiner AP, Edwards TL, Velez Edwards DR (2015). Genetic determinants of pelvic organ prolapse among African American and Hispanic women in the Women's Health Initiative. PLoS One.

[37] Ashikari A, Miyazato M, Kimura R, Oshiro T, Saito S Is collagen type III α-1 polymorphism a risk factor for pelvic organ prolapse in Japanese women? In: International Continence Society, Gothenburg, Sweden, 2019. vol E-Poster presentation. p Abstract 136.

[38] Campeau L, Gorbachinsky I, Stancill J, Rohazinski J, Andersson KE. Characterization of SNPs within the MMP-1 promotor region in women with and without POP. In: International Continences Society Annual Meeting, Glasgow, United Kingdom, August 31, 2011.

[39] Batista NC, Bortolini MAT, Silva RSP, Teixeira JB, Melo NC, Santos RGM, Pepicelli FAA, Castro RA (2020). Collagen I and collagen III polymorphisms in women with pelvic organ prolapse. Neurourol Urodyn.

[40] Chen C, Hill LD, Schubert CM, Strauss JF, Matthews CA (2010). Is laminin gamma-1 a candidate gene for advanced pelvic organ prolapse?. Am J Obstet Gynecol.

[41] Chen Y, Huang J, Hu C, Hua K (2015). Relationship of advanced glycation end products and their receptor to pelvic organ prolapse. Int J Clin Exp Pathol.

[42] Chen HY, Chung YW, Lin WY, Wang JC, Tsai FJ, Tsai CH (2008). Collagen type 3 alpha 1 polymorphism and risk of pelvic organ prolapse. Int J Gynaecol Obstet.

[43] Chen HY, Lin WY, Chen YH, Chen WC, Tsai FJ, Tsai CH (2010). Matrix metalloproteinase-9 polymorphism and risk of pelvic organ prolapse in Taiwanese women. Eur J Obstet Gynecol Reprod Biol.

[44] Chen HY, Wan L, Chung YW, Chen WC, Tsai FJ, Tsai CH (2008). Estrogen receptor beta gene haplotype is associated with pelvic organ prolapse. Eur J Obstet Gynecol Reprod Biol.

[45] Chen J, Li L, Lang J, Zhu L (2020). Common variants in LAMC1 confer risk for pelvic organ prolapse in Chinese population. Hereditas.

[46] Choy KW, Wong ASW, Cheon WC, et al. (2007) Genetic association study in women with pelvic organ prolapse. Paper presented at the International Continence Society, Rotterdam, The Netherlands, August 24, 2007.

[47] Ferrell G, Lu M, Stoddard P, Sammel MD, Romero R, Strauss JF, Matthews CA (2009). A single nucleotide polymorphism in the promoter of the LOXL1 gene and its relationship to pelvic organ prolapse and preterm premature rupture of membranes. Reprod Sci.

[48] Fu R, Hagstrom S, Daneshgari F (2009). Mutation screen of lysl oxidase-like-1 and laminin gamma 1 variant in patients with advanced pelvic organ prolapse. J Urol.

[49] Ghersel FR, Souto RP, Gonzales EWP, Paulo DS, Fernandes CE, Oliveira E (2019). Assessment of metalloproteinase matrix 9 (MMP9) gene polymorphisms risk factors for pelvic organ prolapse in the Brazilian population. Rev Bras Ginecol Obstet.

[50] Jeon MJ, Chung SM, Choi JR, Jung HJ, Kim SK, Bai SW (2009). The relationship between COL3A1 exon 31 polymorphism and pelvic organ prolapse. J Urol.

[51] Karachalios C, Bakas P, Kaparos G, Demeridou S, Liapis I, Grigoriadis C, Liapis A (2016). Matrix metalloproteinase-3 gene promoter polymorphisms: a potential risk factor for pelvic organ prolapse. Biomed Rep.

[52] Kasyan GR, Vishnevskii DA, Akulenko LV, Kozlova YO, Sharova EI, Tupikina NV, Pushkar DY (2017). Association of polymorphism of 1800255 COL3A1 gene with pelvic organ prolapse and urinary incontinence in women: preliminary data. Urologiia.

[53] Khadzhieva MB, Kamoeva SV, Ivanova AV, Abilev SK, Salnikova LE (2015). Elastogenesis-related gene polymorphisms and the risk of pelvic organ prolapse development. Genetika.

[54] Kim JY, Kim EJ, Jeon MJ, Kim R, Lee MW, Kim SW (2014). Association between susceptibility to advanced pelvic organ prolapse and glutathione S-transferase P1 Ile105Val polymorphism. Eur J Obstet Gynecol Reprod Biol.

[55] Kim JY, Kim EJ, Jeon MJ, Kim H, Moon YJ, Bai SW (2014). Association between the poly(ADP-ribose) polymerase-1 gene polymorphism and advanced pelvic organ prolapse. Menopause.

[56] Lince SL, van Kempen LC, Dijkstra JR, IntHout J, Vierhout ME, Kluivers KB (2014). Collagen type III alpha 1 polymorphism (rs1800255, COL3A1 2209 G>a) assessed with high-resolution melting analysis is not associated with pelvic organ prolapse in the Dutch population. Int Urogynecol J.

[57] Maeda PM, Bicudo A, Watanabe RTM, Fonseca TSM, do Souto RP, Fernandes CE, de Oliveira E (2019). Study of the polymorphism rs3025058 of the MMP-3 gene and risk of pelvic organ prolapse in Brazilian women. Eur J Obstet Gynecol Reprod Biol X.

[58] Martins KdF, de Jarmy-DiBella ZIK, A.M.R.M. dF (2011) Evaluation of demographic, clinical characteristics, and genetic polymorphism as risk factors for pelvic organ prolapse in Brazillian women. Neurourol Urodyn 30:1325–1328. 10.1002/nau.21066 21608022

[59] Nakad B, Fares F, Azzam N, Feiner B, Zilberlicht A, Abramov Y (2017). Estrogen receptor and laminin genetic polymorphism among women with pelvic organ prolapse. Taiwan J Obstet Gynecol.

[60] Neupane R, Sadeghi Z, Fu R, Hagstrom SA, Moore CK, Daneshgari F (2014). Mutation screen of LOXL1 in patients with female pelvic organ prolapse. Female Pelvic Med Reconstr Surg.

[61] Palos CC, Timm BF, de Souza PD, Fernandes CE, de Souto RP, Oliveira E (2020). Evaluation of COLIA1-1997 G/T polymorphism as a related factor to genital prolapse. Int Urogynecol J.

[62] Rao S, Lang J, Zhu L, Chen J (2015). Exome sequencing identifies a novel gene, WNK1, for susceptibility to pelvic organ prolapse (POP). PLoS One.

[63] Romero A, Jamison M (2008). Are single nucleotide polymorphisms associated with pelvic organ prolapse?. Pelv Med Surg.

[64] Rosa JPF, Haddad RF, Maeda FGR, Souto RP, Fernandes CE, Oliveira E (2019). Association between col1a2 polymorphism and the occurrence of pelvic organ prolapse in Brazilian women. Rev Bras Ginecol Obstet.

[65] Rusina EI, Bezhenar VF, Ivashchenko TE, Pakin VS, Baranov VS NAT2 , GST T1, GST M1 gene polymorphism and the risk of the development of pelvic organ prolapse and stress urinary incontinence. In: International Continence Society, Rio De Janeiro, Brazil, 2014. vol Poster presentation. p Abstract 258.

[66] Skorupski P, Jankiewicz K, Miotla P, Marczak M, Kulik-Rechberger B, Rechberger T (2013). The polymorphisms of the MMP-1 and the MMP-3 genes and the risk of pelvic organ prolapse. Int Urogynecol J.

[67] Skorupski P, Miotla P, Jankiewicz K, Rechberger T (2010). MMP-1 and MMP-3 gene encoding polymorphism and the risk of the development of pelvic organ prolapse and stress urinary incontinence. Ginekol Pol.

[68] Teixeira FH, Fernandes CE, do Souto RP, de Oliveira E (2020). Polymorphism rs1800255 from COL3A1 gene and the risk for pelvic organ prolapse. Int Urogynecol J.

[69] Vishwajit S, Rohozinski J, Badlani G, Andersson KE Association of MMP1 promoter variant with stress urinary incontinence and pelvic organ prolapse in women. In: International Continence Society Annual Meeting, San Francisco, CA, Oct. 3, 2009 2009.

[70] Wang H, Zhang ZQ, Wang SZ, Lu JL, Wang XL, Zhang ZY (2015). Association of matrix metalloproteinase-10 polymorphisms with susceptibility to pelvic organ prolapse. J Obstet Gynaecol Res.

[71] Wu JM, Visco AG, Grass EA, Craig DM, Fulton RG, Haynes C, Amundsen CL, Shah SH (2012). Comprehensive analysis of LAMC1 genetic variants in advanced pelvic organ prolapse. Am J Obstet Gynecol.

